# Serious Hypokalemia Associated with Abiraterone Acetate in Patients with Castration-Resistant Prostate Cancer

**DOI:** 10.1155/2018/1414395

**Published:** 2018-09-16

**Authors:** Yutaka Yamamoto, Yasunori Akashi, Takahumi Minami, Masahiro Nozawa, Keisuke Kiba, Motokiyo Yoshikawa, Akihide Hirayama, Hirotsugu Uemura

**Affiliations:** ^1^Department of Urology, Nara Hospital, Kindai University Faculty of Medicine, 1248-1 Otodacho Ikoma, Nara 630-0293, Japan; ^2^Department of Urology, Kindai University Faculty of Medicine, 377-2 Ohno-Higashi, Osakasayama, Osaka 589-8511, Japan

## Abstract

**Introduction:**

The treatment strategy for castration-resistant prostate cancer (CRPC) has changed with the approval of several new agents. In 2011, abiraterone acetate was approved for the treatment of metastatic CRPC; however abiraterone is known to cause mineralocorticoid excess syndrome characterized by hypokalemia, fluid retention, and hypertension. We experienced two cases of grade 4 hypokalemia associated with abiraterone treatment.

**Case Presentation:**

Case 1: a 71-year-old male with metastatic CRPC presented with convulsive seizures two weeks after receiving abiraterone plus prednisone. The serum potassium level was 2.1mEq/l. We determined that convulsive seizure was caused by hypokalemia associated with abiraterone. Case 2: a 68-year-old male with metastatic CRPC presented with severe lethargy one month after receiving abiraterone plus prednisone. The serum potassium level was 1.7mEq/l and we concluded that severe lethargy was caused by hypokalemia associated with abiraterone. They were treated with potassium supplementation and increased prednisone following withdrawal of abiraterone.

**Discussion:**

The two patients had been on glucocorticoid therapy before abiraterone therapy. Prolonged administration of exogenous glucocorticoid can lead adrenocortical insufficiency and consequently reduce endogenous glucocorticoid production. This situation may increase the risk of abiraterone-induced mineralocorticoid excess. To reduce the risk of abiraterone-induced hypokalemia, evaluation of adrenocortical insufficiency is required.

## 1. Introduction

Prostate cancer (PCa) is the most common cancer in men in Western industrialized countries [1]. Even though prostate cancer initially responds to androgen deprivation therapy (ADT), most patients with advanced disease invariably develop to castration-resistant prostate cancer (CRPC) within only a few years [2–4]. Recently, the treatment strategy for CRPC has changed with the approval of several new agents. In 2011, abiraterone acetate (abiraterone) was approved by the United States Food and Drug Administration for the treatment of metastatic CRPC. Abiraterone selectively and irreversibly inhibits cytochrome P450 17A1 (CYP17A1) and consequently blocks androgen biosynthesis. Treatment with single-agent abiraterone results in deficient glucocorticoid synthesis and consequently leads to a compensatory upregulation of hypothalamic-pituitary-adrenal (HPA) with raised levels of adrenocorticotrophic hormone (ACTH) [5]. This often leads to an increase of mineralocorticoid production. To prevent mineralocorticoid excess syndrome characterized by hypokalemia, fluid retention, and hypertension, concurrent administration of glucocorticoid is currently adopted in routine practice [6].

Herein, we present two cases of serious hypokalemia associated with abiraterone in patients with metastatic CRPC.

## 2. Case Report

### 2.1. Case 1

**Figure 1:**
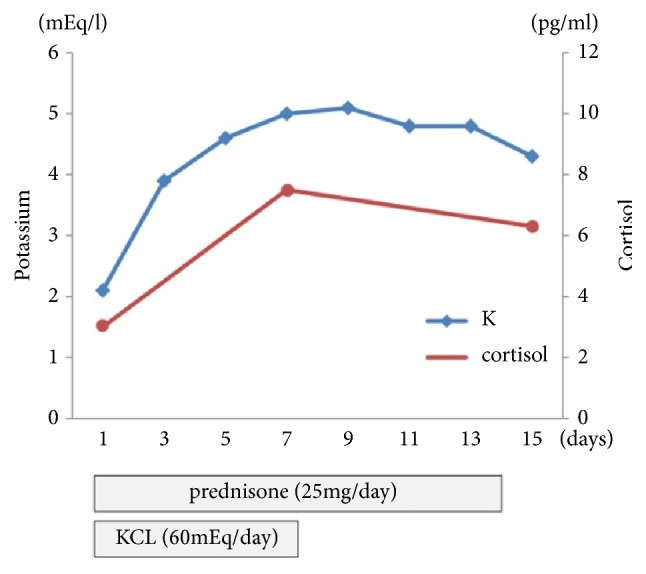
Time-related serum potassium and plasma cortisol changes after prednisone and potassium supplementation.

The patient was a 71-year-old male with metastatic CRPC. At age of 62, he presented with a Gleason score 4+4 adenocarcinoma (cT3aN0M0) and was treated with ADT plus external beam radiation therapy following radical prostatectomy. At age 67, he was diagnosed with CRPC, after PSA levels increased to 11.8 ng/ml and received docetaxel 70 mg/m^2^ with prednisone 10 mg daily. After 16 cycles of docetaxel, the patient presented with biochemical failure, indicated by an elevated PSA level of 40.2 ng/ml. At age 71, abiraterone was given at the standard dose of 1000 mg once daily with prednisone 5 mg twice daily. Two weeks after treatment with abiraterone was initiated, the patient was transferred to Kinki University Hospital, Osaka, Japan, with a chief complaint of convulsive seizures. His blood pressure level was 90/65 and no abnormalities were noted on brain CT. Routine laboratory and endocrinology tests revealed mild liver dysfunction (AST 57 IU/L, ALT 68 IU/L) and decreased levels of potassium 2.1 mEq/l and cortisol 3.0pg/ml (Figure 1). The levels of serum potassium before abiraterone therapy were 4.5mEq/l. We determined that the convulsive seizure occurred as a result of hypokalemia associated with abiraterone therapy. He received potassium supplementation and increased the dose of prednisone to 25 mg/d following discontinuation of abiraterone. Furthermore, furosemide, which was used for a prolonged period because of protracted lower extremity edema, was also interrupted. Seven days after the supplementation therapy, the levels of serum potassium and plasma cortisol were normalized (5.0 mEq/l and 7.5 pg/ml, respectively). He was discharged two weeks after being admitted and was prescribed oral prednisone (20 mg/d).

### 2.2. Case 2

**Figure 2:**
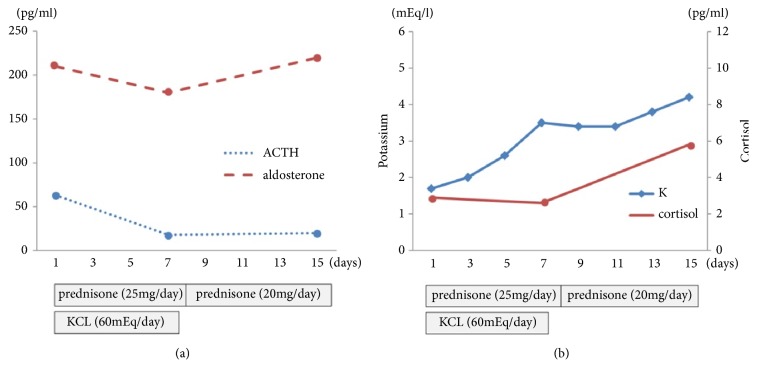
(a) Time-related plasma cortisol and plasma aldosterone changes after prednisone and potassium supplementation. (b) Time-related serum potassium and plasma cortisol changes after prednisone and potassium supplementation.

The patient was a 68-year-old male with metastatic CRPC. At age of 66, he presented with a Gleason score 5+4 adenocarcinoma (cT4N1M1) and was treated with MAB. After 11 months of treatment with MAB, the patient presented a biochemical failure, revealed by an increased PSA value to 10.2 ng/ml. He received docetaxel 70mg/m^2^ with prednisone 10mg daily. However, the treatment was interrupted after 10 months because of severe general fatigue and abiraterone 1000 mg/d with prednisone 5 mg twice daily was initiated. One month after treatment with abiraterone, the patient consulted our hospital with a chief complaint of severe lethargy. His blood pressure was 110/73 and laboratory and endocrinology findings revealed decreased levels of potassium 1.7 mEq/l and cortisol 2.9 pg/ml and elevated levels of ACTH 61.4 pg/ml (Figures 2(a) and 2(b)). Plasma levels of aldosterone were within normal range. The serum level of potassium before abiraterone therapy was 3.2 mEq/l. We established that severe lethargic was caused by hypokalemia associated with abiraterone. This patient also received furosemide for the treatment of chronic heart failure. He received potassium supplementation and increase in prednisone (25 mg daily) following withdrawal of abiraterone and furosemide. Seven days after potassium supplementation therapy, the levels of plasma ACTH and serum potassium were all normalized; however cortisol was still at reference value or lower. At 14 days, plasma cortisol was also normalized and at 20 days after being admitted, the patient was discharged with the use of oral prednisone, 20 mg daily.

## 3. Discussion

**Figure 3:**
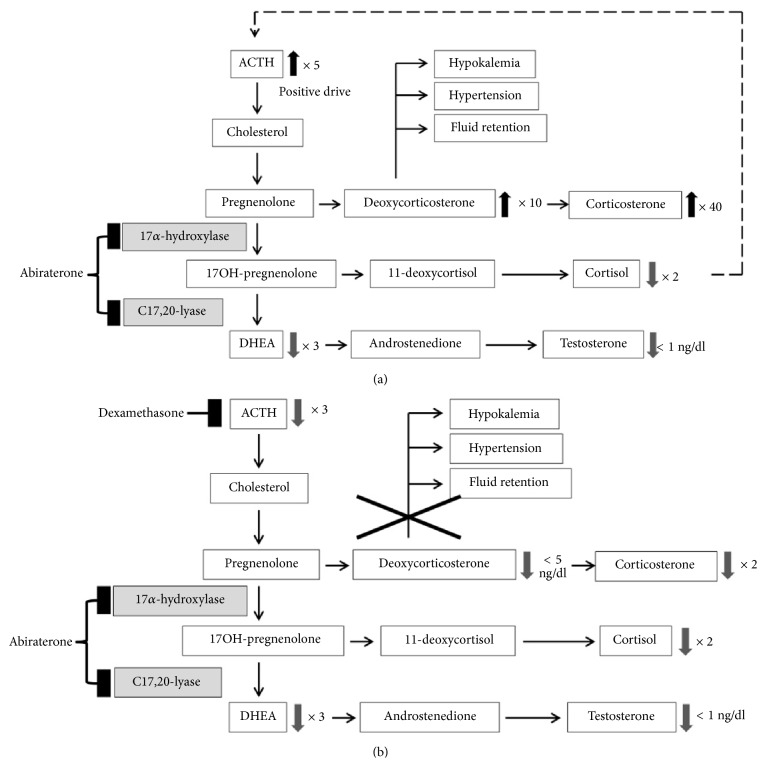
(a) Steroid biosynthesis pathway under abiraterone monotherapy. Inhibition of 17-alpha-hydroxylase and C17 and 20-xylase result in a decrease of cortisol and a consequent increase in ACTH. Increased ACTH causes hypokalemia, fluid retention, and hypertension as a consequence of deoxycorticosterone excess. (b) Steroid biosynthesis pathway under abiraterone plus dexamethasone. Addition of dexamethasone 0.5mg/dl to abiraterone results in suppression of ACTH and a consequent decrease in deoxycorticosterone that prevents hypokalemia, fluid retention, and hypertension. Downstream steroid levels remain suppressed.

Abiraterone is a selective inhibitor of CYP17, which catalyses 17-alpha-hydroxylase and 17, 20-xylase, resulting in a decline of androgen synthesis in adrenal, testis, and prostate cancer tissue [7]. On the other hand, single-agent abiraterone results in suppression of serum cortisol levels by twofold and a consequent increase in ACTH that positively drives the steroid biosynthesis pathway. Up to a fivefold increase in ACTH causes hypokalemia, fluid retention, and hypertension as a consequence of deoxycorticosterone excess (Figure 3(a)). In this setting, adding dexamethasone 0.5 mg/dl to abiraterone suppresses ACTH by threefold, relative to baseline, consequently decreasing deoxycorticosterone to undetectable levels while still maintaining downstream steroid levels suppressed (Figure 3(b)) [8].

Here, we reported two cases of grade 4 hypokalemia associated with abiraterone therapy despite concurrent administration of prednisone at 10 mg/d. Hypokalemia is generally defined as a serum potassium level of less than 3.5 mEq/L. Serum potassium concentrations of 2.5-3.5 mEq/L cause muscle weakness, vomiting, and cataplexy. Serious hypokalemia with potassium level below 2.5 mEq/L often manifests as limb paralysis, cardiac arrhythmias, and acute respiratory failure. Therefore, serious hypokalemia should be detected and managed immediately.

We surmise that the cases involving serious hypokalemia described in this report were provoked by two distinct mechanisms. It is likely that hypokalemia was manifested due the long-term use of glucocorticoids which were coadministered with docetaxel prior to abiraterone. Prolonged administration of exogenous glucocorticoids can lead to suppression of the HPA axis which induces secondary adrenocortical insufficiency consequently reducing endogenous glucocorticoid production [9, 10]. In this setting, treatment with abiraterone plus 10mg of prednisone without additional glucocorticoid supplementation may lead to feedback to the HPA axis, resulting in an increase of ACTH release. Increased ACTH leads to over production of desoxycorticosterone which ultimately manifests as hypokalemia. Following this hypothesis, prednisone at 10mg daily cannot normalize the abiraterone-induced rise in ACTH. Indeed, the patient in case 2 showed decrease of cortisol 2.9 pg/ml and elevated ACTH 61.4 pg/ml at the onset of hypokalemia even though 10mg prednisone was coadministered. We speculate hypokalemia which may result from adrenocortical insufficiency due to prolonged administration of exogenous glucocorticoid coadministered with docetaxel. Two large prospective randomized phase III trials (COU-AA-301 and COU-AA-302 trials) tested the efficacy and safety of abiraterone plus prednisone versus placebo plus prednisone in the postchemotherapy and prechemotherapy settings [11, 12]. These studies showed that the incidence of adverse events related to mineralocorticoid excess was higher in the abiraterone group than the placebo group. Furthermore, all-grade hypokalemia related to abiraterone was mostly the same in both studies (16.6 % in the prechemotherapy and 18.0 % in the postchemotherapy). However, an apparently higher incidence of grade 3-4 hypokalemia related to abiraterone was observed in COU-AA-301 but not in COU-AA-302 (4.4% of abiraterone arm and 0.8% of control in the COU-AA-301, 2.6% of abiraterone arm, and 1.9% of control in the COU-AA-302). Taken together, these results indicate that the incidence of high grade hypokalemia related to abiraterone tends to occur among postchemotherapy patients than prechemotherapy patients. The question now is how should one proceeds when starting abiraterone therapy for patients who had received systemic glucocorticoid therapy? Long-term exposure to glucocorticoid produces a variety of adverse events including increased risk of hyperglycemia, bone metabolism, immunosuppression, and cognitive impairment [13–17]. Adrenocortical insufficiency has been also recognized as glucocorticoid related AEs [10, 18]; however, the dosing period and dosage of glucocorticoid which affect the risk of adrenocortical insufficiency are unclear. Some reported a positive relationship between them [19] and others a negative [20]. Thus, consistent with above-mentioned hypothesis, measuring plasma ACTH and cortisol before abiraterone therapy should be determined especially for patients who are more sensitive to abiraterone as a consequence of adrenocortical insufficiency.

The two patients in this report demonstrated that potassium and prednisone supplementation are efficacious in controlling hypokalemia associated with abiraterone. Meanwhile, treatment with mineralocorticoid receptor antagonist should also be considered as another option. The mineralocorticoid receptor antagonists, spironolactone or eplerenone, are currently approved for the management of heart failure and primary hypertension [21, 22]. However, spironolactone interacts with androgen receptor [23, 24] and therefore cannot be recommended for CRPC patients. In contrast, eplerenone was used to inhibit mineralocorticoid excess in earlier clinical studies of abiraterone [8, 25, 26]. Although further clinical trials used prednisone or other low dose glucocorticoids, eplerenone remains as a useful option to relieve secondary mineralocorticoid excess by abiraterone in the real-world setting [24, 27]. Currently, there is no consensus as to whether a glucocorticoid or mineralocorticoid receptor antagonist is a better choice for controlling mineralocorticoid excess. It may be preferable to add eplerenone when glucocorticoid supplementation is insufficient to manage abiraterone-induced hypokalemia.

The other possible reason for the manifestation of hypokalemia described in this report is due to concurrent administration of furosemide, a drug known to cause hypokalemia. Furosemide acts on the ascending limb of the loop of Henle to inhibit Na-K-Cl symporter resulting in low potassium levels [28]. The two patients in this report were also taking furosemide. Although it is unclear whether drug-drug interactions between abiraterone and furosemide could augment the risk of hypokalemia, switching from furosemide to other diuretics should be considered before treatment with abiraterone. In this regard, potassium-sparing diuretic eplerenone may be preferable to prevent hypokalemia. Therefore when a diuretic is needed, switching from furosemide to eplerenone should be considered from the beginning of the abiraterone therapy.

In conclusion, these case reports demonstrate that treatment with abiraterone and furosemide instigated serious hypokalemia in patients with CRPC. Hypokalemia is easily manageable with appropriate monitoring and is less critical than the AEs associated with cytocidal therapies, yet serious hypokalemia often provokes arrhythmia, flaccid paralysis, and respiratory depression, which are life-threatening complications that require urgent treatment. The severity of mineralocorticoid related AEs following abiraterone seems to differ on an individual basis. To reduce the risk of hypokalemia, evaluation of adrenocortical insufficiency and concomitant drugs is required. This unique hypothesis needs to be elucidated in the future trials.
